# Récidive d’une pseudo-exfoliation capsulaire sur implant intraoculaire

**DOI:** 10.11604/pamj.2018.30.47.10864

**Published:** 2018-05-18

**Authors:** Bennis Ahmed, Benatiya Andaloussi Idriss

**Affiliations:** 1 Résidence Sara, Prestigia, Fes City Center, Champs de Course, Fès, Maroc; 2 Faculté de Médecine et de Pharmacie de Fès CHU Hassan II, Fes, Maroc

**Keywords:** Pseudo-exfoliation capsulaire, implant intraoculaire, glaucome exfoliatif, Capsular exfoliation, intraocular implant, exfoliative glaucoma

## Abstract

We here report the case of a 79-year old patient who underwent cataract by phacoemulsification associated with preoperative left and right eyes pseudo-capsular exfoliation in 2000. He had pseudo-exfoliative glaucoma with recurrence on the implant.

## Image en médecine

Patient de 79 ans, opéré de cataracte par phaco-émulsification en 2000, avec une pseudo-exfoliation capsulaire ODG préopératoire. Présente un glaucome pseudo-exfoliatif avec une récidive sur l'implant.

**Figure 1:**
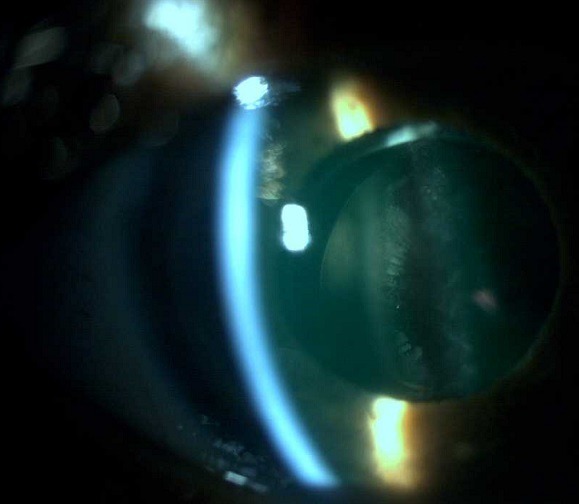
Présence d’une récidive de pseudo-exfoliation capsulaire sur l’implant intraoculaire, chez un patient opéré depuis 16 ans par une phaco-emulsification avec mise en place d’un implant dans le sac capsulaire

